# PD75 Evolution Of Health Technology Submissions In The Unified Health System: Analysis Of National Committee For Health Technology Incorporation (CONITEC) Evaluations (2012 to 2024)

**DOI:** 10.1017/S0266462325102055

**Published:** 2025-12-29

**Authors:** Aíla Carmo, Denis Satoshi Komoda, Luciana Xavier, Luciene Bonan, Nayara Brito, Priscila Louly

## Abstract

**Introduction:**

The establishment of health technology assessment (HTA) in health systems is essential for their sustainability. In Brazil, CONITEC was legally established in 2011, and its first reports were published in 2012. CONITEC has consistently contributed to maintaining evidence-based access to technologies in Brazil’s Unified Health System (SUS).

**Methods:**

This study aimed to summarize and understand the characteristics of submitted requests such as type of technology, tendencies, and deliberation outcomes. All reports for technologies submitted to CONITEC that were available on the official website, published from 2012 to November 2024, were included. Data of interest included: year of assessment, nature of the request (inclusion, disinvestment, or expansion of use to subgroups not already indicated for the technology), type of technology (medication, procedure, or health product), and medical field (e.g., oncology, rheumatology). Absolute and relative frequencies as well as temporal analyses were performed on the extracted data.

**Results:**

From a total of 1,084 (average of 90.3 per year) identified reports, 836 were related to medications, 173 to products, and 75 to procedures. Inclusion, disinvestment, and expansion accounted for 928 (85.6%), 89 (8.2%), and 63 (5.8%) of the reports, respectively. A growth trend in the number of reports per year was not observed, despite a peak in 2021 (115 assessments). This peak was due to a concentration of reports on disinvestment, which represented 31.3 percent of the reports conducted that year. The three most represented medical fields were infectiology (14.2%), oncology (11.5%), and hematology (7.2%).

**Conclusions:**

The large number of HTA reports assessed by CONITEC showed the potential of the Brazilian SUS to promptly respond to health needs. Further studies may reveal why incorporation and disinvestment are the most frequently submitted requests. Monitoring requests can help understand the dynamics of HTA, which is crucial for system management and sustainability.

